# Acute electromyostimulation Decreases Muscle Sympathetic Nerve Activity in Patients with Advanced Chronic Heart Failure (EMSICA Study)

**DOI:** 10.1371/journal.pone.0079438

**Published:** 2013-11-12

**Authors:** Marc Labrunée, Fabien Despas, Philippe Marque, Thibaut Guiraud, Michel Galinier, Jean Michel Senard, Atul Pathak

**Affiliations:** 1 National Institute of Health and Medical ResearchTeam 8 Institut des maladies métaboliques et cardiovasculaires, Toulouse, France; 2 Faculty of Medicine, University of Toulouse III Paul Sabatier F-31432, Toulouse, France; 3 Federation of Cardiology, Universitary Hospital of Toulouse, F-31073, Toulouse, France; 4 Physical Medicine and Rehabilitation unit, Universitary Hospital of Toulouse, F-31073, Toulouse, France; 5 Clinical Pharmacology unit, Universitary Hospital of Toulouse, F-31073, Toulouse, France; 6 Cardiopulmonary rehabilitation center, Saint-Orens de Gameville, France; University of Adelaide, Australia

## Abstract

**Background:**

Muscle passive contraction of lower limb by neuromuscular electrostimulation (NMES) is frequently used in chronic heart failure (CHF) patients but no data are available concerning its action on sympathetic activity. However, Transcutaneous Electrical Nerve Stimulation (TENS) is able to improve baroreflex in CHF. The primary aim of the present study was to investigate the acute effect of TENS and NMES compared to Sham stimulation on sympathetic overactivity as assessed by Muscle Sympathetic Nerve Activity (MSNA).

**Methods:**

We performed a serie of two parallel, randomized, double blinded and sham controlled protocols in twenty-two CHF patients in New York Heart Association (NYHA) Class III. Half of them performed stimulation by TENS, and the others tested NMES.

**Results:**

Compare to Sham stimulation, both TENS and NMES are able to reduce MSNA (63.5 ± 3.5 vs 69.7 ± 3.1 bursts / min, p < 0.01 after TENS and 51.6 ± 3.3 vs 56.7 ± 3.3 bursts / min, p < 0, 01 after NMES). No variation of blood pressure, heart rate or respiratory parameters was observed after stimulation.

**Conclusion:**

The results suggest that sensory stimulation of lower limbs by electrical device, either TENS or NMES, could inhibit sympathetic outflow directed to legs in CHF patients. These properties could benefits CHF patients and pave the way for a new non-pharmacological approach of CHF.

## Introduction

Chronic heart failure (CHF) is characterised by sympathetic overactivity causing direct effect on the initiation and progression of heart failure. Consequently, sympathetic activity (SA) is a strong predictor of morbidity and mortality [1]. Risk –related to this feature are numerous. Among them, the risk of sudden death, but also muscle weakness leading to exercise intolerance are common[2].. Thus, SA represent a direct or indirect target for most therapeutics used in CHF as beta-blockade drugs [3] or resynchronization therapy [4,5].

It has been shown that exercise can improve symptoms, morbidity and outcomes related to CHF partly due to a diminution of resting SA [6,7]. Exercise techniques used in this setting are time consuming, costly and cannot be well applied to severe CHF patients. Neuromuscular electrical stimulation (NMES) could be an alternative in these patients [8–10]. Indeed, the repetition of NMES on lower limbs is known to improve muscular atrophy with specific increase of muscular oxidative fibres (type I), allowing better aerobic capacity [11–13]. In CHF patients, some studies shows that NMES modulates immunity and improve blood flow and muscle functioning [14] Beside these peripheral effects due to passive muscular contraction, NMES also induces a sensory stimulation. In healthy subjects, cutaneous electrical stimulation has an inhibitory effect on sympathetic activity [15]. In CHF patients, it has also been shown recently that cutaneous electrical stimulation improved baroreflex sensitivity [16] and authors hypothesized that TENS could interact with sympathetic activity. However, in this study, patients were not randomized, the study was not controlled (i.e no sham stimulation) and SA was not measured. 

We therefore decided to undertake the following study in order to demonstrate that TENS benefits (i.e. baroreflex sensitivity enhancement) could be related to a direct effect on SA as assessed by Muscle Sympathetic Nerve Activity (MSNA). In addition, since NMES, unlike TENS, is the electrical standard treatment used in the rehabilitation of CHF patients, we sought to test whether NMES would be associated with a decrease in SA (TENS effect during NMES) or another modulation of sympathetic activity.

Using a double blind, randomized, sham controlled design, we examined successively the effects of TENS and NMES on sympathetic activity assessed directly by nerve recording (MSNA) in CHF patients.

## Methods

### Ethics statements

Twenty two patients (all in New York Heart Association (NYHA) Class III) with systolic CHF were prospectively recruited. All patient received pharmacotherapy according to the current guidelines for advanced CHF corresponding to Beta-blockade drugs, Angiotensin-converting enzyme inhibitors or angiotensin II type-1 receptor inhibitors, diuretics and anti-aldosterone drugs. Exclusion criteria were: non sinusal rhythm, severe cardiac arrhythmia, diabetes, sensibility deficiency, neuropathy, chronic pain on leg. Informed written consent was obtained from all participants in accordance with standards established by the latest revision of the Declaration of Helsinki. The study was approved by the local Institutional Human Subjects Review Committee named “CPP Sud-Ouest et Outre Mer II”.

### Measurements

Heart rate (HR) was measured continuously by an electrocardiogram (ADInstruments, Castle Hill, New South Wales, Australia). Blood Pressure was measured continuously by the Finometer system (Finometer, Finapress Medical SystemBV, Amsterdam, The Netherlands). Multiunit postganglionic sympathetic nerve activity was recorded as previously described [17]. Briefly, a tungsten microelectrode (shaft diameter 200mm, tapering to an uninsulated tip of 1–5mm) was inserted selectively into muscle or skin fascicles of the fibular nerve. A subcutaneous reference electrode was first inserted 2–3 cm away from the recording electrode, which was itself inserted into the nerve fascicle. The neural signals were amplified, filtered, rectified and integrated to obtain a voltage display of sympathetic nerve activity. The intralaboratory reproducibility of microneurography has been assessed previously [17]. 

Each burst was carefully determined, and the sympathetic activity was calculated as bursts per minute and bursts for 100 hearts beats (which allows comparison of sympathetic discharge between individuals) by a blinded investigator (ML) in both protocols for baseline and post stimulation (TENS and NMES).

All patients had a standard cardiac transthoracic echocardiography with assessment of classical parameters among them the left ventricular ejection fraction estimated by Simpson estimation.

### Protocol and procedures

The both protocols were performed in parallel because of the difficulty in carrying out too long assessment in heart failure patients who are frail and tired and to limit the risk of loss of MSNA signal over time. However, each of the two protocols (A and B) was cross over, randomized and sham controlled. Thus, in consequence with these methodological particularities, the 22 subjects were randomized either to TENS (Protocol A) or NMES (Protocol B) but not to both of them. Subjects were in a supine rest position for thirty minutes before MSNA recording, at the same time of the day. For each patient, the total duration of participation in the protocol was about 3 hours. We tested the effect of two different electrical stimulations. TENS is classically able to induce stimulation of big myelinated afferent fibers, property used for pain treatment [18]. NMES is able to induce stimulation of efferent fibers causing muscular contraction which is the objective of NMES, for example in CHF patient. 

#### Protocol A (aimed to assess the effects of cutaneous stimulation on SA, through TENS procedure).

Patient had solely skin stimulation without muscular contraction, through the use of TENS procedure. Current intensity was defined as the maximum level of current able to induce sensory stimulation without muscular contraction. The electrodes were placed in the left leg in front of the quadriceps and the suralis triceps. The current, delivered by an electrostimulator (Physio 4, CEFAR©, Sweden), was non polarised with a frequency of 80 Hz, a pulse width at 200us. The total duration of stimulation was of five minutes alternating with 3 seconds of stimulation and 3 seconds of rest. The intensity was equal to the maximum to produce sensory stimulation without muscle contraction. The Sham stimulation shared the same characteristics that TENS except for intensity that was increased to reach the minimal threshold able to induce cutaneous sensation without any muscular contraction, as the Sham method described in the reference study [13]. Because of electrical interferences, MSNA could not be recorded simultaneously during electrostimulation. The two sets of stimulation (TENS and Sham) were administrated in a random order. MSNA was recorded in the right leg immediately after electrical stimulation cessation. Blood pressure, heart rate and the respiratory parameters (respiration rate, oxygen saturation) were recorded during all the procedure.

#### Protocol B (aimed to assess the effects of both muscular and cutaneous stimulation on SA, through NMES procedure).

Patient had a muscular and sensory stimulation by NMES. As in protocol A, electrodes were placed in the left leg in front of the quadriceps and the suralis triceps. A non polarised current was administrated by an electrostimulator (Physio 4, Cefar,

Sweden) during five minutes, with a frequency at 25 Hz, an intensity to obtain maximal tolerated muscular contraction, and a pulse width at 200 µs. The total duration of stimulation was five minutes alternating with 3 seconds of stimulation and 3 seconds of rest. The Sham stimulation shared the same characteristics that NMES except for intensity that was increased to reach the minimal threshold able to induce cutaneous sensation without any muscular contraction. The two sets of stimulation (NMES and Sham) were made in a random order. MSNA was recorded in the right leg immediately after electrical stimulation cessation. Because of electrical interferences, MSNA cannot be recorded simultaneously during electrical stimulation. Blood pressure, heart rate and the respiratory parameters (respiration rate, oxygen saturation) were recorded during all the procedure.

### Sample size

Our primary endpoint was the effect of acute stimulation on MSNA, as measured by bursts per minute. Based on our previous studies realized on similar type of patients (namely CHF patients), we hypothesized that one session of TENS or NMES would be able to modify the MSNA by 25%. This value is approximately what we observed in studies where where comorbidities of heart failure modulate sympathetic reflexes (i.e anemia [19], chronic kidney disease or cardio-renal syndrome [20]). Moreover this is 50% of the effect induced by chronic exercise on MSNA in a rehabilitation program realized in chronic heart failure patients [7]. The repeated measure approach has been used as method of comparison in a crossover type study (with a correlation coefficient equal to 0,5, an alpha coefficient equal to 5%, a power equal to 90% and a bilateral hypothesis). Hence, the sample size calculation induced a number of subjects equal to 11 for each of the protocols A and B.

### Analyses

Demographic data and baseline characteristics of the two groups (Protocol A versus Protocol B) were compared by the use of an unpaired student’s t test and chi-square tests with Yates correction. Data of protocol A and B were analysed by the use of a repeated measure ANOVA for parameters obtained during and after stimulation with post hoc analysis (Sidak correction). Data are presented as the mean ± SEM. A *p* value less than 0.05 was considered significant with a bilateral hypothesis.

## Results

### Population study

The clinical characteristics of the patients are noted in table 1. There were no significant differences between the two groups regarding clinical features.

**Table 1:** Clinical characteristics of patients.

**Clinical characteristics of patients**	**Protocol A, n = 11**	**Protocol B, n = 11**	***p***
**Age (year)**	62.7 ± 3.6	54.4 ± 3.8	NS
**Men / Women**	7 / 4	7 / 4	NS
**BMI (kg/m^2^)**	27.4 ± 1.8	27.1 ± 1.1	NS
**Ischemic heart disease (%)**	64	64	NS
**NYHA class**	III (11)	III (11)	NS
**Echography LVEF (%)**	24 ± 2.8	30 ± 3.6	NS
**Resting MSNA (burst / min)**	68.3 ± 2.9	57.3 ± 2.8	NS
**Treatments**			
**β-blockers**	10 (91%)	11 (100%)	NS
**ACE inhibitor or AT1 receptor blockers**	10 (91%)	10 (91%)	NS
**Diuretics**	10 (91%)	9 (82%)	NS
**Anti aldosterone**	7 (64%)	8 (73%)	NS

**Values are mean ± SEM; NYHA, New York Heart Association; LVEF, Left Ventricular Ejection Fraction; ACE, Angiotensin-Converting Enzyme; AT1, Angiotensin II type-1; MSNA, Muscle Nerve Sympathetic Activity.**

#### Protocol A (aimed to assess the effects of solely cutaneous stimulation on SA, through TENS procedure).

**Figure 1:**
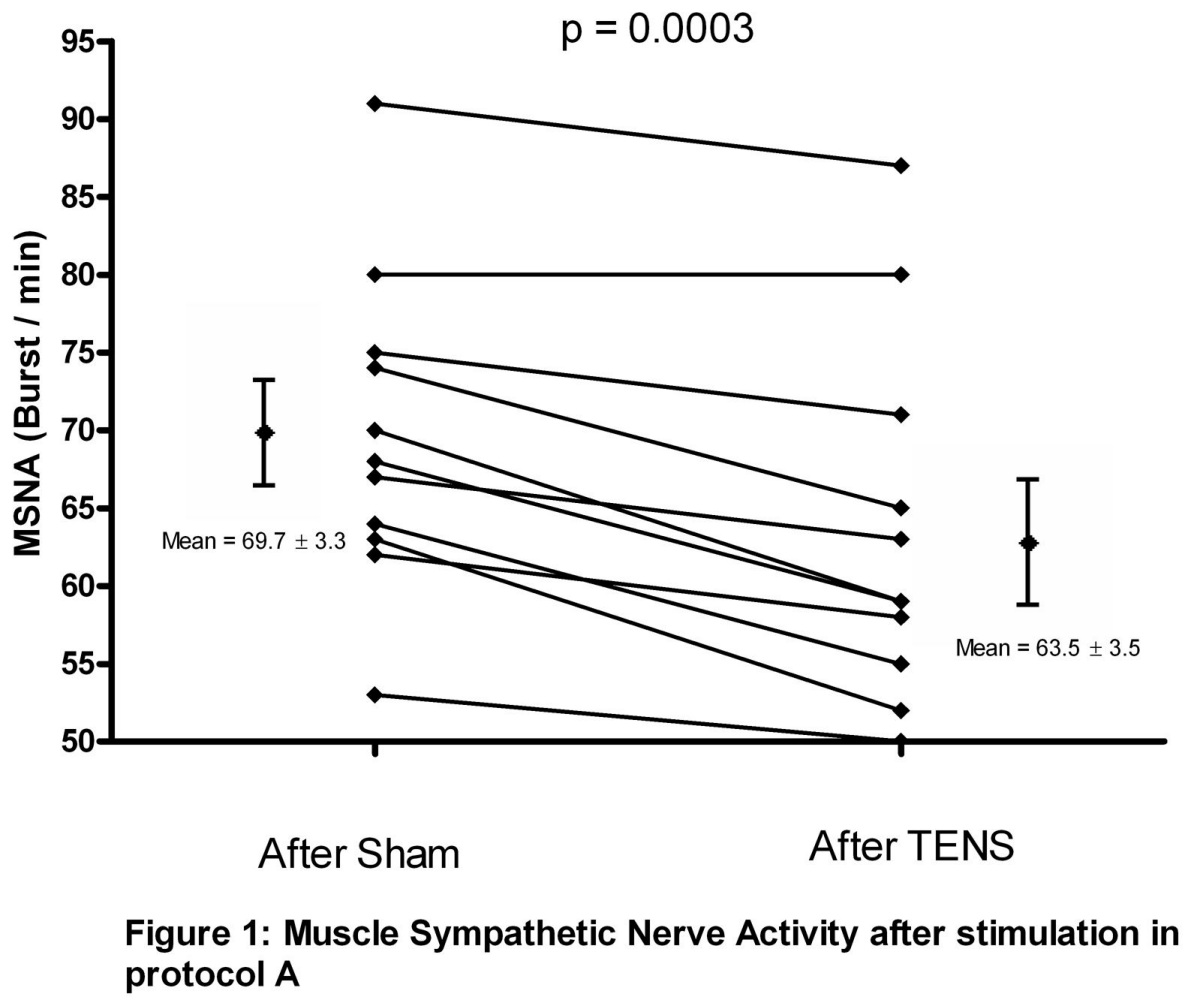
Muscle Sympathetic Nerve Activity after stimulation in protocol A.

Eleven patients were randomized to perform the protocol A. During the electrical stimulation (table 2), heart rate, blood pressure and respiratory parameters were unchanged. During the first minute post TENS stimulation (table 2 and Figure 1), there was a significant decrease in MSNA compared with the period after Sham stimulation (63.5 ± 3.5 vs 69.7 ± 3.1 burst.min^-1^, p < 0.01, 81.1± 5.3 vs 87.8± 4.1 burst.100 HB^-1^ delta of -9.1 ± 1.6 %). Blood pressure, heart rate and the respiratory parameters were not changed. 

**Table 2:** Parameters in protocol A.

**Measurements**	**Baseline**	**Sham (1st minute)**	**Sham (5th minute)**	**Post sham**	**TENS (1st minute)**	**TENS (5th minute)**	**Post TENS**	**ANOVA (p)**
**MSNA (burst.min^-1^)**	**67.8±3.1**	**-**	**-**	**69.7 ± 3.1**	**-**	**-**	63.5 ± 3.5 *	< 0,05
**MSNA (burst.100 HB^-1^)**	**89.5±4.9**	**-**	**-**	**87.8 ± 4.1**	**-**	**-**	81.1 ± 5.3 *	< 0,05
**SBP (mmHg)**	**109.2±4.6**	**105.2± 4.7**	**105.1 ± 3.7**	**106.3 ± 4.4**	**108.9 ± 5.5**	**104,6 ± 5.3**	**106.2 ± 4.6**	ns
**DBP (mmHg)**	**67.1±4.1**	**65.2± 4.2**	**65.6 ± 3.3**	**67.1 ± 3.4**	**65.6 ± 4.0**	**63.9 ± 4.0**	**64.2 ± 3.4**	ns
**HR (beats.min^-1^)**	**78.0±3.9**	**78.1± 3.5**	**79.2 ± 3.3**	**80.4 ± 3.3**	**78.3 ± 3.3**	**77.1 ± 3.0**	**80.5 ± 4.7**	ns
**Respiration rate (cycle.min^-1^)**	**17.6±0.9**	**17.9±1.2**	**17.6±1.1**	**17.4±0.9**	**17.7±0.9**	**17.5±1.0**	**17.4±0.8**	ns
**Oxygen Saturation (%)**	**96±0.7**	**96.9±0.6**	**96.9±0.6**	**96.2±0.8**	**96.7±0.6**	**96.6±0.6**	**96.2±0.9**	ns

Values are mean ± SEM, MSNA, Muscle Nerve Sympathetic Activity; HB, Heart Beat; SBP, Systolic Blood Pressure; DBP, Distolic Blood Pressure; HR, Heart Rate; TENS, Transcutaneous Electric Nerve Stimulation. * for p<0.05 in post hoc analysis (Post sham Vs post TENS).

#### Protocol B (aimed to assess the effects of both muscular and cutaneous stimulation on SA, through NMES procedure).

**Figure 2:**
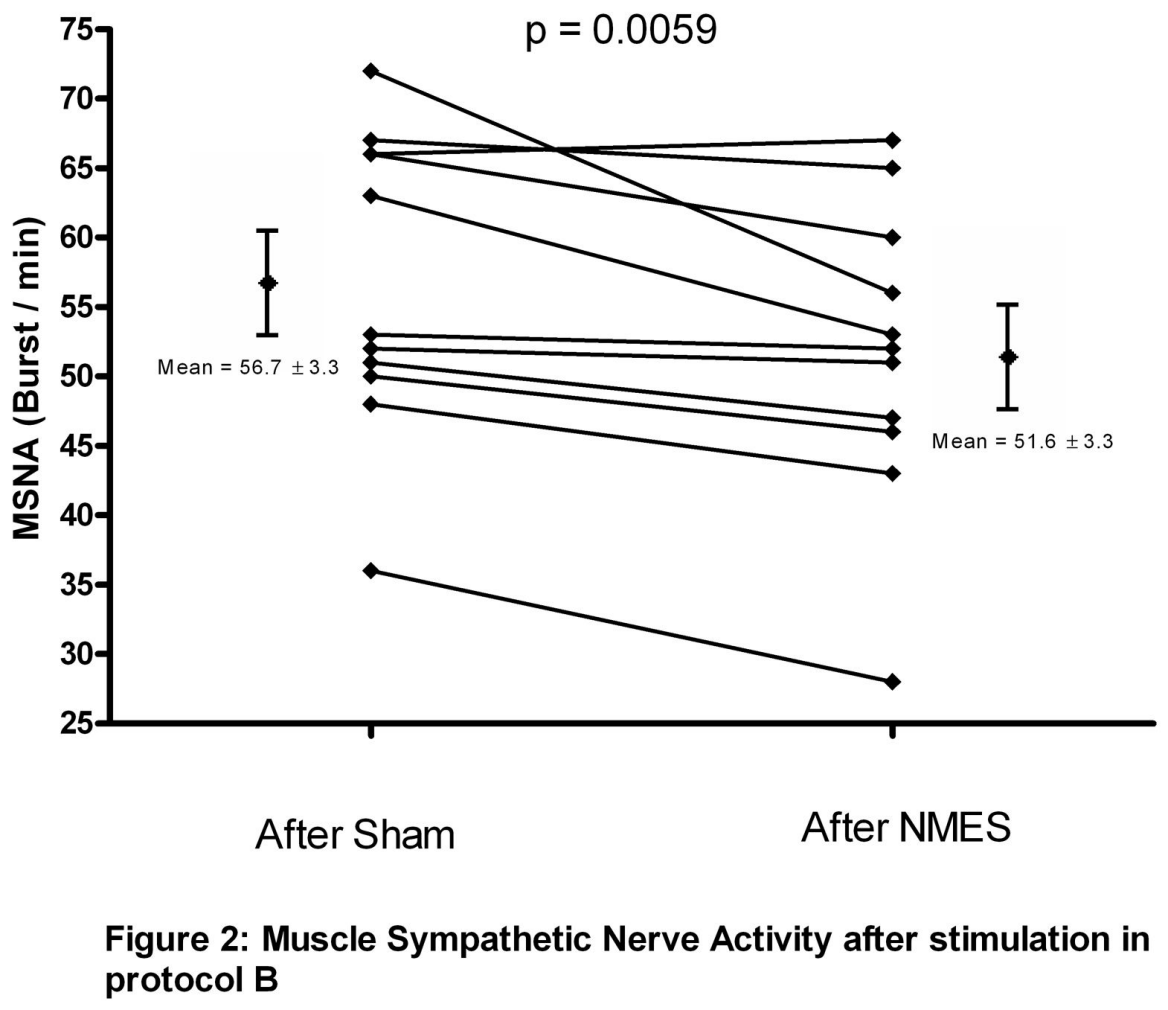
Muscle Sympathetic Nerve Activity after stimulation in protocol B.

**Figure 3:**
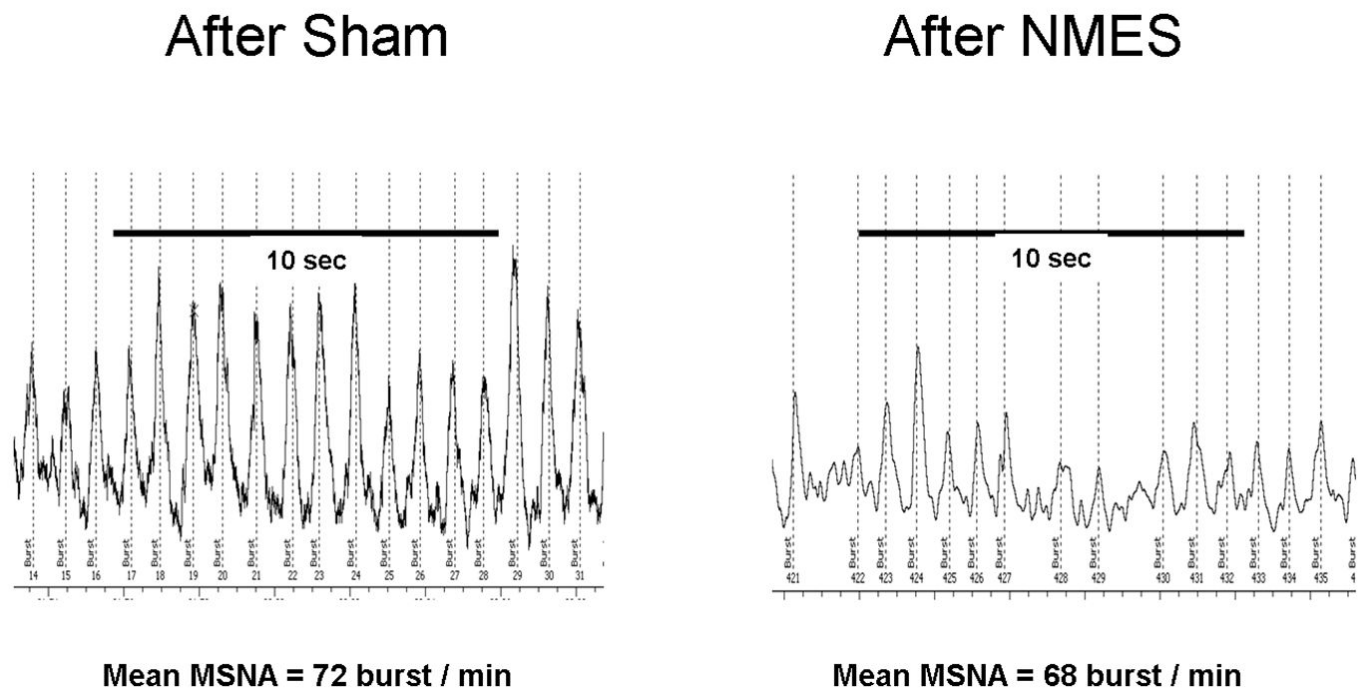
example of Muscle Sympathetic Nerve Activity after sham stimulation and after NeuroMuscular Electrical Stimulation in one patient (protocol B).

Eleven patients were randomized to perform protocol B. During stimulation (NMES), heart rate, blood pressure and respiratory parameters were unchanged (table 3). After NMES stimulation, MSNA was significantly decreased in comparison to the period after Sham stimulation (51.6 ± 3.3 vs 56.7 ± 3.3 burst.min^-1^, p < 0, 01; 72,2 ± 3,9 vs 80,9 ± 3,9 burst.100 HB^-1^, delta of -9.2 ± 2.4 % ) without difference in blood pressure, heart rate and the respiratory parameters (table 3, figure 2 and 3). 

**Table 3:** Parameters in protocol B.

**Measurements**	**Baseline**	**Sham (1st minute)**	**Sham (5th minute)**	**Post Sham**	**NMES (1st minute)**	**NMES (5th minute)**	**post NMES**	**ANOVA (p)**
**MSNA (burst.min^-1^)**	**56±3.7**	**-**	**-**	**56.7 ± 3.3**	**-**	**-**	51.6 ± 3.3*†	<0.05
**MSNA (burst.100 HB^-1^)**	**81.7±3.6**	**-**	**-**	**80.9 ± 3.7**	**-**	**-**	72.2 ± 3.9*†	<0.05
**SBP (mmHg)**	**105.8±4.6**	**108.7± 4.1**	**110.8 ± 5.0**	**107.5 ± 5.0**	**109.0 ± 4.8**	**110,1 ± 4.8**	**107.9 ± 4.4**	ns
**DBP (mmHg)**	**57.3±2.8**	**57.2± 2.2**	**58.8 ± 2.5**	**56.3 ± 2.9**	**57.5 ± 2.2**	**59.6 ± 2.5**	**57.2 ± 2.0**	ns
**HR (beats.min^-1^)**	**70.8±3.7**	**70.4± 3.7**	**71.5 ± 4.2**	**70.8 ± 4.3**	**70.8 ± 4.2**	**71.3 ± 4.4**	**70.8 ± 4.3**	ns
**Respiration rate (cycle.min^-1^)**	**16.6±1.6**	**17.1±1.3**	**16.7±1.4**	**16.3±1.4**	**17.2±1.3**	**17.9±1.4**	**16.5±1.3**	ns
**Oxygen Saturation (%)**	**95.4±0.8**	**94.1±1.1**	**93.8±1.0**	**94±1.2**	**93.8±1.1**	**94.6±1.2**	**94.6±1.1**	ns

Values are mean ± SEM, MSNA, Muscle Nerve Sympathetic Activity; HB, Heart Beat; SBP, Systolic blood pressure; DBP, Distolic blood pressure; HR, heart rate; NMES, NeuroMuscular Electrical Stimulation. * for p<0.05 in post hoc analysis (Post sham Vs post NMES), † for p<0.05 in post hoc analysis (baseline vs post NMES).

## Discussion

In this double blind, randomized and sham-controlled study, we provide for the first time evidence that TENS is able to directly reduce sympathetic activity in patients with CHF. We also show that this putative beneficial effect linked to a sensory stimulation is preserved while CHF patients are exposed to an electromyostimulation by NMES, the latter combining sensory and muscular stimulation.. 

To the best of our knowledge, this is the first time that sympathetic activity was measured by MSNA after lower limb electrical stimulation in CHF patients. MSNA is considered as the most accurate and sensitive technique comparatively to more standard techniques such as catecholamine dosage [21,22] or heart rate variability [23] to assess sympathetic activity and its fast changes during a protocol. The elevated SA known in CHF is correlated to symptoms, progression of heart failure and prognosis [24,25]. This induces vasoconstriction and therefore reduces tissue perfusion as the heart or peripheral muscle causing cardiac arrhythmia, muscle fatigue and so a limitation of physical ability. The study of Barretto et als. [1] showed that MSNA has an independent prognostic value in heart failure patients with a threshold value of 49 bursts per minute. In their study, MSNA was a better parameter able to predict mortality than other biochemical, hemodynamic or cardiac functional predictors of outcome in CHF. Consequently decreasing MSNA could be considered as a key target of any intervention in CHF. Hence, reduction of MSNA induced by therapeutics like beta-blockade drugs [3], resynchronisation [5] or exercise [7] is associated with enhancement of functional capacity. Our results suggest that electrical stimulation of the lower limbs (TENS or NMES) decrease MSNA to the same extent , this underlines the importance of this type of treatment. NMES was mainly known to induce muscular effects [8,13,14] but the effects on the autonomic nervous system were unknown. Our study helps to better understand the potential benefits and the use of this novel approach in CHF leading to a reduction of muscle peripheral vasoconstriction, even though our protocol evaluates a single stimulation rather than repeated stimulation over several weeks, as it is usually done. Finally, in our study, the differences observed in MSNA between groups were unlikely to be influenced by differences in drug therapies between groups thus emphasizing the specific effect of lower limb electrical stimulation on MSNA.

### 1– Effect of TENS (sensory stimulation) on sympathetic baroreflex-dependent mechanisms

We first show that TENS is able to reduce SA as assessed by microneurographic recording. This is the first time that effect of cutaneous stimulation on SA, measured directly in CHF patient, is realized. The diminution of MSNA is not due to a local axonal reflex which could be implicated in alteration of the efferent vegetative fibers response directed to lower limbs [26] because in our study, MSNA is recorded in the limb opposite to stimulation. So reduction of MSNA could be rather attributable to central nervous system alteration (either in the brain or spinal cord). The cutaneous stimulation induced by TENS in CHF patients is able to enhance spontaneous baroreflex sensitivity, considered as a surrogate marker for autonomic function [16]. The authors hypothesized the role of muscle afferents fibers stimulation induced by TENS (ergoreflex) on liberation of substance P on Nucleus Tractus Solitary [27] that could interact with baroreflex sensitivity [28] which is linked with sympathetic outflow. However, this study was not randomized and patients were matched toward an exercise arm and not a real control Sham group. Finally, the evaluation of the sympathetic activity was only performed indirectly through spontaneous baroreflex analysis. It is well established that this technique informs more about the vagal response rather than on the sympathetic control of cardiovascular parameters. In clinical situations other than CHF, there are several arguments for MSNA reduction after skin stimulation. In healthy subjects, skin stimulation by a brief electrical impulse is known to reduce amplitude of the first two bursts of MSNA immediately after stimulation [15]. In our study, using direct assessment of sympathetic baroreflex function, we provide a confirmation of Gademan’s hypothesis [16] suggesting that TENS could decrease SA via an improvement of baroreflex sensitivity.

### 2– Effect of TENS (sensory stimulation) on sympathetic baroreflex-independent mechanisms

Another mechanism could explain our results in addition of the modulation of baroreflex. Electroacupuncture, a form of cutaneous electrical stimulation apparented to TENS, is known to inhibit sympathetic activity via opioïd excretion in spinal cord [29,30]. Furthermore, direct stimulation of spinal cord in the sensitive dorsal funiculus by electric impulse induces vasodilatation on legs and can be used in case of ischemia due to peripheral artery disease [31]. These results suggest that afferent nerve stimulation by electrical device could directly act on medullar component of sympathetic nervous system. Indeed, in this protocol, we predominantly realized a stimulation of skin nerve fibers since TENS current is known to be preferentially conducted by large myelinated fibers rather than unmyelinated or small afferent fibers [32–34]. Hence, this possible medullar action of TENS is probably able to alleviate the sympathetic tone towards local organs (skin, vessels etc…) and could therefore explain the decrease of MSNA (table 2).

### 3– Effect of NMES (muscular contraction + sensory stimulation)

In our study, NMES (protocol B) is associated with a significant decrease of MSNA (decrease of 9.2 % towards baseline) with no modifications of hemodynamic or respiratory parameters similarly to the results shown in protocol A (TENS effect). These results are surprising, since non electrical muscle stimulation is usually associated with an increase of sympathetic outflow in CHF patients in response to activation of afferents nerve fibers type III and IV. This is related to both, muscular contraction or stretch, known to induce an elevation of MSNA [35]. However, the mechanism of the SA’s increase after muscular stimulation is still a matter of controversies, while some authors state that local release of metabolites can lead to an increase of SA [36] , others have shown that in CHF patient, metaboreceptors are not activated [37]. One study performed in healthy volunteers has shown that active muscular stimulation by handgrip leads to a sympathoexcitation assessed by MSNA [38]. Additionally, a study making NMES in healthy volunteers, showed an increase of SA through indirect assessment of sympathetic activity using blood pressure as a surrogate [39]. In CHF patients, the effects of NMES on SA have never been investigated. Our study is the first to provide this description of sympathoinhibitory effect related to NMES. When comparing the intensity of muscle stimulation, NMES is less intense than passive or active contraction, this should lead to slight increase of MSNA but not to a reduced SA as seen in our study. This in turn suggest that reduction of SA by NMES is related to another pathways involving other triggers than the muscle and taking into account central integration as we have discussed previously with TENS in protocol A. Hence, our study open the path for the concept that NMES beside beneficial muscular effect is also able through a “TENS effect” to reduce sympathetic activity known to be a marker of the progression and complications of heart failure patients. If NMES induces muscular contraction conducted by motor efferent fibers to muscle (sympatho excitatory effect), it also induces skin sensory stimulation (similar to TENS stimulation) under the electrode (sympathoinhibitory effect) which is probably predominant in our protocol.

### 4– Effect of TENS or NMES on hemodynamic and respiratory parameters

In our study, despite reduction of MSNA we didn’t find significative variation of blood pressure and / or heart rate. These results are in accordance with Gademan’s observation [16]. This is probably due to : i) the baroreflex desadaptation in CHF patients [40,41]; ii) the moderated change of MSNA; iii) the mode of stimulation. In addition, we found no variation for respiratory parameters hence avoiding a potential bias to interpret MSNA modification during the protocol.

### 5– Limits

We were unable to record continuously MSNA due to interferences between electrical stimulation and microneurography recording. The potential consequence of this technical limitation is that during stimulation, the modulation of MSNA has not been evaluated. However hemodynamic and respiratory parameters were not modified during stimulation suggesting that MSNA was not influenced by stimulation during this acute phase. 

Other parameters like catecholamine level or heart rate variability could have been recorded during stimulation but both are indirect measurements of SA and thus less powerful than MSNA. Finally it is well known that other reflexes could influence SA modulation (such as chemo, metabo or mechano-reflexes) but we did not analyse these pathways in our study. We also cannot exclude local release of substance under electrode that could be at the origin of MSNA modification by modulation of endothelial function as suggested by Karavidas in a repetitive NMES stimulation [42].

### 6– Perspectives

In this cross over randomized study we show for the first time that electromyostimulation in CHF patients is able to reduce muscle sympathetic nerve activity partly through a somatosensory stimulation. These data suggest that in CHF patient unable to perform physical activity this type of approach could provide both muscular rehabilitation and putatively enhancement of the natural history of CHF since SA has been considered as a risk factor for cardiovascular complications. These acute effects need to be confirmed by a long-term approach. This refers to a clinical trial in which we are currently involved (referenced as NCT 01548508 on clinicaltrials.gov) aiming to demonstrate that NMES could improve CHF patient status through SA decrease.
